# Antibacterial and Antibiofilm Activities of Novel Antimicrobial Peptides against Multidrug-Resistant Enterotoxigenic *Escherichia Coli*

**DOI:** 10.3390/ijms22083926

**Published:** 2021-04-10

**Authors:** Kang-Chi Wu, Kuo-Feng Hua, Yu-Hsiang Yu, Yeong-Hsiang Cheng, Ting-Ting Cheng, Yao-Kuan Huang, Hui-Wen Chang, Wei-Jung Chen

**Affiliations:** 1 Department of Biotechnology and Animal Science, National Ilan University, Yilan 26047, Taiwan; c82630@gmail.com (K.-C.W.); kuofenghua@gmail.com (K.-F.H.); yuyh@niu.edu.tw (Y.-H.Y.); yhcheng@ems.niu.edu.tw (Y.-H.C.); la860726@gmail.com (T.-T.C.); ddt1124@gate.sinica.edu.tw (Y.-K.H.); 2 Institute of Cellular and Organismic Biology, Academia Sinica, Taipei 11529, Taiwan; 3 Department of Veterinary Medicine, School of Veterinary Medicine, National Taiwan University, Taipei 10617, Taiwan; huiwenchang@ntu.edu.tw; 4 Graduate Institute of Molecular and Comparative Pathobiology, School of Veterinary Medicine, National Taiwan University, Taipei 10617, Taiwan

**Keywords:** enterotoxigenic *Escherichia coli* (ETEC), antimicrobial peptides (AMPs), multidrug resistance (MDR), antibiofilm, intestinal porcine epithelial cell-1 (IPEC-1)

## Abstract

Post-weaning diarrhea due to enterotoxigenic *Escherichia coli* (ETEC) is a common disease of piglets and causes great economic loss for the swine industry. Over the past few decades, decreasing effectiveness of conventional antibiotics has caused serious problems because of the growing emergence of multidrug-resistant (MDR) pathogens. Various studies have indicated that antimicrobial peptides (AMPs) have potential to serve as an alternative to antibiotics owing to rapid killing action and highly selective toxicity. Our previous studies have shown that AMP GW-Q4 and its derivatives possess effective antibacterial activities against the Gram-negative bacteria. Hence, in the current study, we evaluated the antibacterial efficacy of GW-Q4 and its derivatives against MDR ETEC and their minimal inhibition concentration (MIC) values were determined to be around 2~32 μg/mL. Among them, AMP Q4-15a-1 with the second lowest MIC (4 μg/mL) and the highest minimal hemolysis concentration (MHC, 256 μg/mL), thus showing the greatest selectivity (MHC/MIC = 64) was selected for further investigations. Moreover, Q4-15a-1 showed dose-dependent bactericidal activity against MDR ETEC in time–kill curve assays. According to the cellular localization and membrane integrity analyses using confocal microscopy, Q4-15a-1 can rapidly interact with the bacterial surface, disrupt the membrane and enter cytosol in less than 30 min. Minimum biofilm eradication concentration (MBEC) of Q4-15a-1 is 4× MIC (16 μg/mL), indicating that Q4-15a-1 is effective against MDR ETEC biofilm. Besides, we established an MDR ETEC infection model with intestinal porcine epithelial cell-1 (IPEC-1). In this infection model, 32 μg/mL Q4-15a-1 can completely inhibit ETEC adhesion onto IPEC-1. Overall, these results suggested that Q4-15a-1 may be a promising antibacterial candidate for treatment of weaned piglets infected by MDR ETEC.

## 1. Introduction

Post-weaning diarrhea (PWD) due to *Escherichia coli* (*E. coli*) is one of the most serious problems for the swine industry worldwide. PWD is usually associated with the proliferation of enterotoxigenic *E. coli* (ETEC) in the pig intestine, characterized by diarrhea and dehydration, causing more than 50% of piglets to die [1,2,3], thus resulting in considerable economic losses in the swine industry. Although vaccination and management measures are available, antibiotics may still be required in some cases [4]. Antibiotics commonly used to treat swine diseases caused by *E. coli* include apramycin, gentamicin, neomycin, sulfa drugs, and enrofloxacin [4]. The antibacterial efficacy of most antibiotics is to interfere with the bacterial structure or its metabolic process. Common mechanisms of action are: inhibition of cell wall synthesis, destruction of cell membrane structure or function, inhibition of nucleic acid structure and function, inhibition of protein synthesis, and blockage of key metabolic pathways [5,6]. However, extensive use of traditional antibiotics has led to the growing emergence of many resistant strains of pathogenic bacteria, including ETEC. Therefore, the development of novel therapeutic agents that could conquer the resistance problem has become a crucial issue.

It has been suggested that antimicrobial peptides (AMPs), also called host defense peptides (HDPs), are of greatest potential to serve as antibiotic substitutes [7,8,9]. AMPs belong to the innate immune system and host defense mechanism of a wide range of living organisms [7,8,9]. However, bacteria have developed a variety of efficient resistance mechanisms against AMPs, such as secretory proteases, alterations of the bacterial cell surface composition, and efflux pumps [10,11]. Therefore, we have designed and synthesized a series of cationic and amphipathic α-helical AMPs with enhanced activity and selectivity against a broad spectrum of Gram-positive and Gram-negative strains [12]. According to our previous studies, our novel AMPs exhibited potent antibacterial efficacy against several marine pathogenic bacteria [13,14,15,16] and another important bacterial pathogen in pigs, *Salmonella enterica* serovar Choleraesuis [17]. However, it remains unknown whether AMPs possess antibacterial activity to combat multidrug-resistant (MDR) strains.

Generally, bacteria do not live freely in suspension (planktonic cells), but in complex communities called biofilms. Biofilms are defined as bacterial communities enclosed in a self-produced extracellular polymeric matrix that are attached to a biotic or abiotic surface [18,19,20]. Biofilms protect bacteria from several stress conditions and challenges including extreme environments, the immune response, antibacterial agents and disinfectants [21]. Bacterial colonization results from two distinct physiological processes, namely bacterial adhesion and biofilm formation. Broadly speaking, a biofilm is defined as the sessile development of microbial cells [22]. ETEC colonizes the small intestine of piglets, where diarrhea is caused. Upon entering the host, ETEC bacteria must pass through the acidic environment of the stomach; overcome the bactericidal effects of bile in the duodenum, jejunum, and ileum; and traverse the mucous layer of the small intestine before colonizing the epithelial surface and establishing an infection. Biofilm formation may also play an important role during the process. Recent studies have also demonstrated that the antimicrobial resistance of ETEC is associated with biofilm formation.

In this study, we intended to explore the antibacterial and anti-biofilm activities of our self-designed novel AMPs against MDR strains of ETEC using MIC, MBC, and MBEC assays. Moreover, we also established an MDR ETEC infection model with IPEC-1 cell line. Findings provided in this study could help us to understand the possible reaction mechanism of these novel AMPs and may facilitate the development of promising antibacterial candidate for treatment of weaned piglets infected by MDR ETEC.

## 2. Results

### 2.1. Design of Truncated and Substituted AMP Derivatives

**Figure 1:**
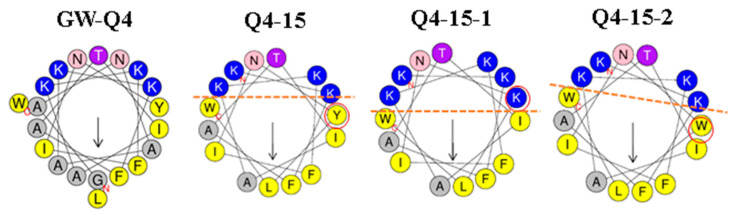
Helical-wheel diagrams of antimicrobial peptides (AMPs) using HeliQuest (http://heliquest.ipmc.cnrs.fr/ accessed on 1 March 2021). Positively charged residues (Lys) are shown in blue, Asn in pink, and Thr in magenta. Hydrophobic residues (Trp, Tyr, Phe, Leu, Ile) are shown in yellow, Gly and Ala are shown in gray. The arrows indicated the orientation of hydrophobic moment.

According to our previous studies, AMPs GW-Q4, and GW-Q6 were reported to be the most potent ones against a broad spectrum of bacterial strains, especially Gram-negative bacteria [12]. In this study, GW-Q4 was selected since it exhibited strongest antibacterial activity against *Escherichia coli* [13]. In order to obtain a shorter peptide with at least equivalent antibacterial efficacy, several truncations from N- and C-terminus were performed (data not shown), and the N-terminal truncated peptide Q4-15 resulted to be the best among all derivatives. To obtain a novel peptide with better antibacterial activity and selectivity, a Tyr residue located on the interface of hydrophilic and hydrophobic phases were selected and substituted with Lys (Q4-15-1) or Trp (Q4-15-2), respectively. C-terminal amide derivatives for all AMPs were also prepared to increase the net positive charge. The amino acid sequence, molecular weight and structural parameters for all AMPs used in the current study were summarized in Table 1. Helical-wheel diagrams of AMPs were plotted using HeliQuest (http://heliquest.ipmc.cnrs.fr/ accessed on 1 March 2021) and shown in Figure 1.

### 2.2. Q4-15a-1 Showed the Highest Therapeutic Index (TI) against MDR ETEC

The antibacterial and hemolytic activity of these AMPs against MDR ETEC were thus evaluated and their minimum inhibitory concentration (MIC), minimum hemolytic concentration (MHC) and therapeutic index (TI) values were listed in Table 2. The MICs of AMPs were measured to be 2–32 μg/mL, and the MHCs were 16–256 μg/mL, resulting in TI (MHC/MIC) values of 4–64. Higher TI values indicated greater selectivity. Among them, AMP Q4-15a-1 with the second lowest MIC (4 μg/mL) and the highest MHC (256 μg/mL), thus showing the greatest selectivity (TI = 64) was selected for further investigations.

### 2.3. Q4-15a-1 Exhibited Rapid Bactericidal Effect against MDR ETEC

**Figure 2:**
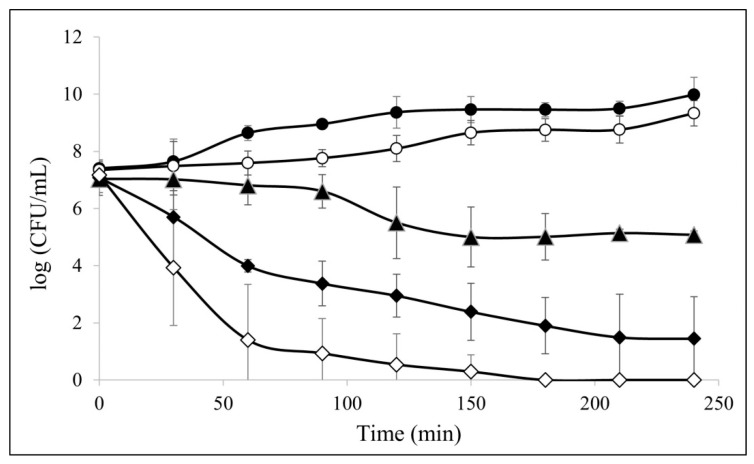
Time–kill curves of multidrug-resistant enterotoxigenic *Escherichia coli* (MDR ETEC) strain treated with different concentration of Q4-15a-1. (1× MIC = 4 μg/mL) 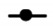 Untreated; 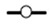 0.5× MIC; 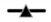 1× MIC; 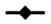 2× MIC; 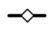 4× MIC.

The ability of Q4-15a-1 in time killing MDR ETEC was further evaluated by analyzing the fractional cell survival upon AMP treatment at 0.5×, 1×, 2×, and 4× MIC concentrations. As shown in Figure 2, the bacteria were completely killed upon exposure to the peptide for an hour at 4× MIC concentration. Thus, decreasing killing efficiency were also found for the two strains at 1× and 2× MIC. According to our results, Q4-15a-1 had a dose-dependent and rapid bactericidal effect against MDR ETEC.

### 2.4. Q4-15a-1 Showed Potent Antibiofilm Activity against MDR ETEC

Since Q4-15a-1 showed the best antibacterial selectivity against MDR ETEC, we then investigated if it could avoid the formation of biofilm in 96-well plates by calculating the minimum biofilm eradication concentration (MBEC) using the Calgary Biofilm Device. The term MBEC is defined as the minimum concentration needed to inhibit the re-growth of biofilms after 24 h of peptide treatment [24]. Viable biofilm cells of MDR ETEC after treatment with Q4-15a-1 of various concentrations were determined and listed in Table 3. In this study, the MBEC value was calculated to be 15.93 ± 4.07 μg/mL (Table 4). When incubated with 4 μg/mL of AMP for 24 h, the remaining viable biofilm cells were 1.32%, while 16 μg/mL of AMP resulted in a viable biofilm cells of 0.003%. The results indicated that Q4-15a-1 was able to inhibit the biofilm formation (> 99.9%) at a minimum concentration of 16 ± 4 μg/mL, that is, the MBEC and MBECb values of Q4-15a-1 against MDR ETEC were both around 16 ± 4 μg/mL, which is only 4× MIC. Here we provided evidence for the first time that our AMP possess potent antibiofilm activity against MDR bacterial strains.

### 2.5. Localization of Q4-15a-1 in MDR ETEC

**Figure 3:**
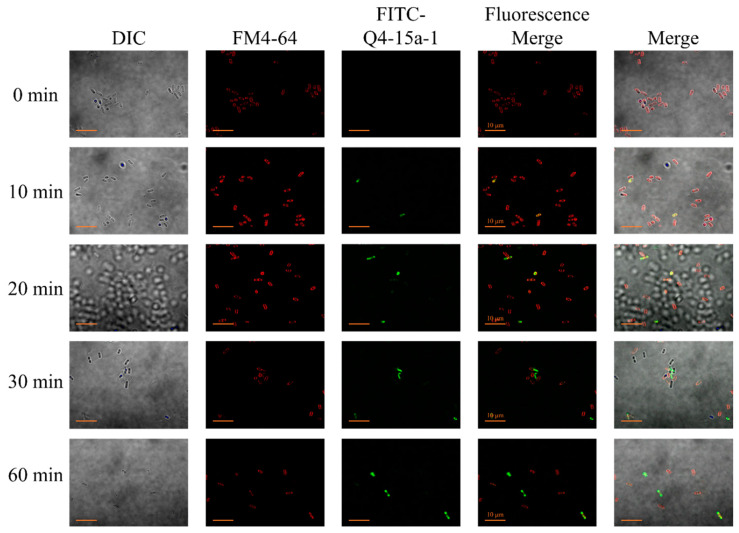
Localization of FITC-Q4-15a-1 in multidrug-resistant enterotoxigenic *Escherichia coli* (MDR ETEC) at different time intervals monitored by confocal microscopy. Bar: 10 μm.

Before observing whether Q4-15a-1 can damage bacterial cell membranes, we first monitored the localization of Q4-15a-1 in MDR ETEC by confocal microscopy. The experiment was carried out by using a red-fluorescent FM4-64, which is a bacterial cell membrane-specific dye, and the green fluorescent FITC-labeled Q4-15a-1. As shown in Figure 3, when MDR ETEC was treated with FITC-Q4-15a-1 for 10–20 min, the yellow signals in the merged images indicated that the peptide was first attached to the bacterial surface and co-localized with the cell membrane. After 30 min, the red and green signals were clearly separated, suggesting that Q4-15a-1 started to enter the bacteria and located in the cytoplasm. The results revealed that Q4-15a-1 can rapidly interact with bacterial surface and penetrate cell membrane within 30 min.

### 2.6. Q4-15a-1 Caused Bacterial Cell Membrane Damage in MDR ETEC

**Figure 4:**
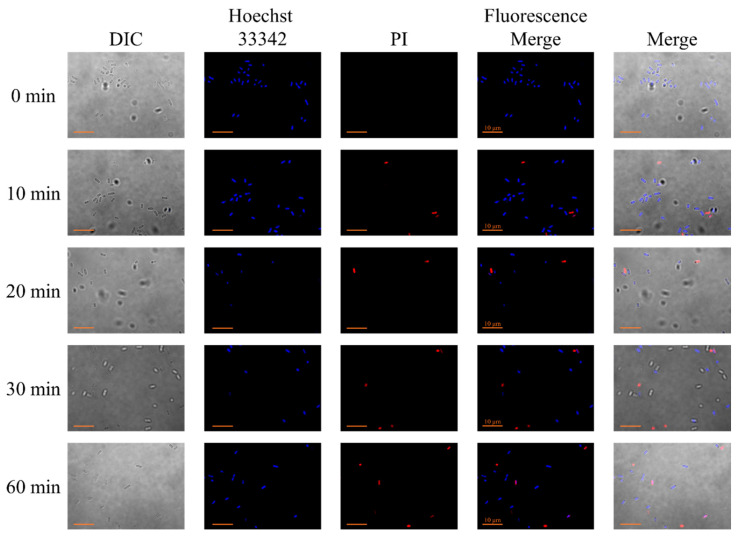
Cell membrane integrity of MDR ETEC treated with Q4-15a-1 of 4× MIC (16 μg/mL) for 10, 20, 30, or 60 min monitored by confocal microscopy. Bar: 10 μm.

In order to verify whether Q4-15a-1 can cause bacterial cell membrane damage, we also performed confocal microscopy by utilizing two fluorescent DNA intercalators, Hoechst 33,342 and Propidium iodide (PI). Since Hoechst can readily cross cell membranes to stain DNA of living and dead cells, it was used to label the total number of bacterial cells. In contrast, PI only enters cells with compromised plasma membranes, thus selectively labels dead cells. As shown in Figure 4, the membrane-permeable Hoechst 33,342 is a minor groove-binding DNA stain that emits bright blue fluorescence upon binding to DNA. The blue fluorescence can be clearly seen with or without treatment of Q4-15a-1. While the red fluorescence of PI staining can only be monitored when MDR ETEC was treated with Q4-15a-1 for 10–60 min. The pink signals in the merged images indicated the co-localization of both DNA dyes. The results suggested that Q4-15a-1 did interact and interfere with the bacterial cell membrane integrity of MDR ETEC in a short period of incubation time.

### 2.7. Q4-15a-1 Caused Bacterial Cell Membrane Damage in MDR ETEC

**Figure 5:**
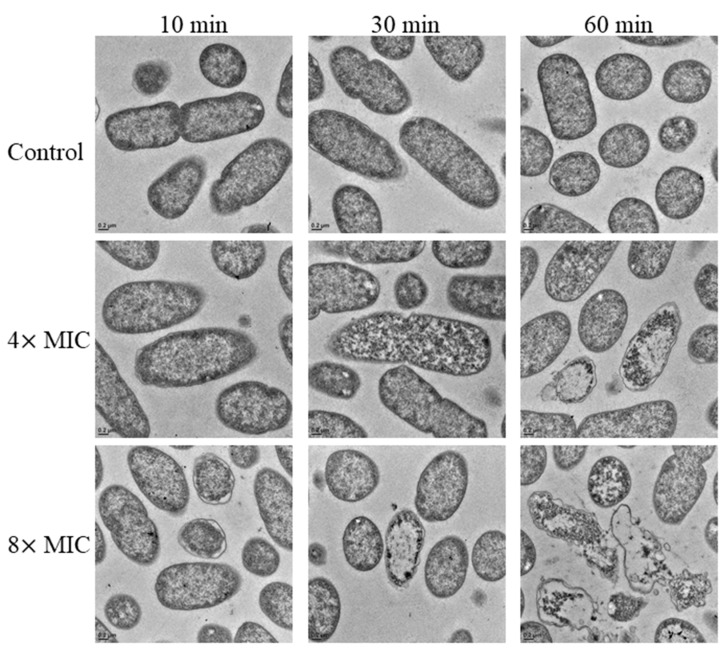
Transmission electron microscopy images of 2 × 10^9^ CFU/mL of MDR ETEC left untreated (control) or treated with Q4-15a-1 of 4× or 8× MIC (16 or 32 μg/mL) for 10, 30, or 60 min. Scale bars, 0.2 μm.

To visualize how AMP Q4-15a-1 interact with *E. coli*, transmission electron microscopy (TEM) studies were performed (Figure 5). Overall, non-treated bacteria had intact membranes and an even intracellular distribution of DNA and ribosomes in the cytoplasm (light and darker areas, respectively). After 30 min, exposure to 32 μg/mL (8× MIC) Q4-15a-1 resulted in release of membrane vesicles and caused intracellular changes, such as clustering of DNA and ribosome condensations compared to non-treated bacteria. After treatment for 60 min, Q4-15a-1 compromised membrane integrity and increased ribosome clustering and cell lysis.

### 2.8. Q4-15a-1 Inhibited the Adherence of MDR ETEC onto IPEC-1 Cells

**Figure 6:**
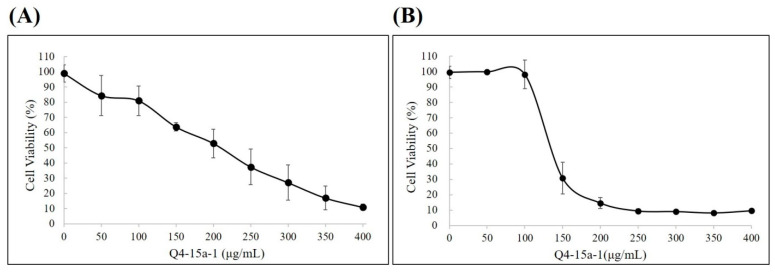
Cell viability of (**A**) intestinal porcine epithelial cell line-1 (IPEC-1) and (**B**) intestinal porcine epithelial cell line-J2 (IPEC-J2) upon exposure to different concentrations of AMP Q4-15a-1.

We then tried to evaluate whether AMP Q4-15a-1 could inhibit the infection of porcine intestinal cells by ETEC. In this study, two porcine small intestinal epithelial cell lines (IPEC-1 and IPEC-J2) were applied. Before performing the infection test, we wanted to confirm whether AMP Q4-15a-1 would cause damage to the above two cell lines. MTT assay was conducted and the cell viability results were shown in Figure 6. The IC_20_ of Q4-15a-1 against IPEC-1 and IPEC-J2 is around 100 μg/mL, that is, AMP Q4-15a-1 is not harmful to both cell lines while remain effective against ETEC.

**Figure 7:**
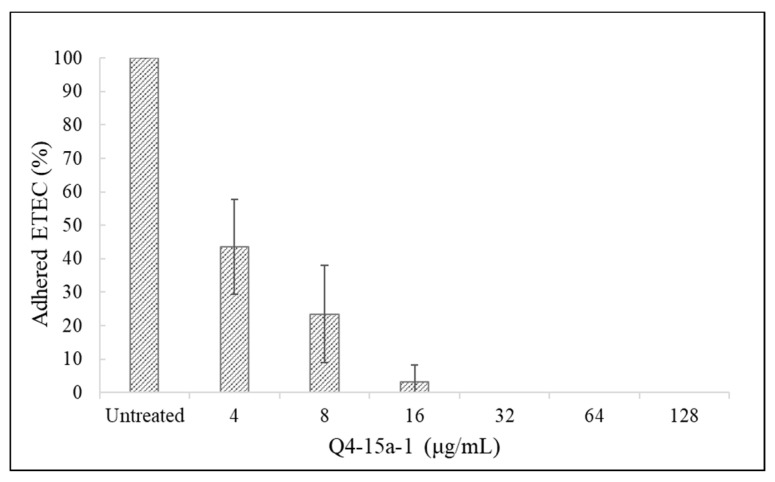
Inhibitory effect of Q4-15a-1 against the adherence of MDR ETEC to IPEC-1 cells.

The MDR ETEC strain used in this study has been determined to be F18-ciliated. According to a previous report indicating that IPEC-1 is more susceptible to F18 (K88) ciliated ETEC infection, and is less likely to adhere to IPEC-J2 cell lines [25,26], IPEC-1 cell line was selected for the following adherence assay. In order to test whether Q4-15a-1 can reduce and inhibit the adherence of MDR ETEC to IPEC-1 cells, Q4-15a-1 and ETEC were added to IPEC-1 cells at different concentrations and co-cultured. We diluted the plated bacteria solution 3 h after infection and calculated the relative ETEC adherence rate. The results are shown in Figure 7. When ETEC and 4 μg/mL Q4-15a-1 were co-cultured at the same time, the adhered ETEC was reduced to 43% of the original untreated group, and as the dose of Q4-15a-1 increased, the adherence rate significantly decreased. When ETEC were co-cultured with Q4-15a-1 of more than 32 μg/mL, the adherence of ETEC to IPEC-1 cells was completely inhibited.

## 3. Discussion

The present study was to explore the antibacterial and anti-biofilm activities of our self-designed novel AMPs against MDR strains of ETEC using MIC, MBC, and MBEC assays. Furthermore, we also established an MDR ETEC infection model with intestinal porcine epithelial cell-1 (IPEC-1). Our findings in this study revealed that AMP Q4-15a-1 showed minimal cytotoxicity in the IPEC-1 cell line, while it exerted strong antimicrobial activity on MDR ETEC and significantly reduced its adhesion to IPEC-1 cells. Findings provided in this study could help us to understand the possible reaction mechanism of our novel AMPs and may facilitate the development of promising antibacterial candidate for treatment of weaned piglets infected by MDR ETEC.

In view of the increasing emergence of MDR *E. coli* and the lack of alternative treatments for infectious diseases [27], it is necessary to develop alternative methods to control the spread of this pathogen and homologous infections. Evidence has suggested that AMPs are of greatest potential to represent such a new type of antibiotics [3,4,5]. We have designed and developed a series of cationic α-helical AMPs with potent activity and selectivity against a broad spectrum of Gram-negative and Gram-positive strains [12,13,14,15,16]. In our previous study, a potent AMP GW-Q6 exhibited potential antimicrobial activity against an MDR strain of *Salmonella enterica* serovar Choleraesuis [17]. Proteomics methodologies were applied to delineate the stress response mechanisms of the MDR pathogens against our novel AMPs. However, it remained unclear whether these novel AMPs possess antibacterial and anti-biofilm activity to combat MDR ETEC. In this study, we aimed to optimize our novel AMPs with better antibacterial activity and selectivity, and evaluate their potential against MDR ETEC, another important bacterial pathogen in the swine industry.

Based on our previous reports, GW-Q4 was shown to be the most potent AMP against *Escherichia coli* [12,13]. In the current study, we performed truncations and modifications of GW-Q4 in order to obtain shorter peptide fragments with better antibacterial activity and selectivity. Among the 8 AMPs obtained, Q4-15a-2 exhibited the lowest MIC value of 2 μg/mL (Table 2), indicating the best antibacterial activity. However, its rather low MHC value (16 μg/mL) is indicative of strong hemolytic activity, which resulted in the TI (MHC/MIC) of only 8, a rather poor selectivity. Thus, Q4-15a-1, which showed the second lowest MIC value of 4 μg/mL and MHC value of 256 μg/mL (Table 2), was selected for subsequent investigations because of its highest antibacterial selectivity (TI = 64). Previous studies have shown that interfacial membrane interactions between AMPs and bacteria are crucial to their biological functions and activities [28]. A balance of positive charge, hydrophobicity and amphipathicity (hydrophobic moment) is required for high antimicrobial activity and selectivity [12].

It has been reported that AMPs can show a rapid bactericidal effect on microorganisms because they can directly damage bacterial cell membranes [29,30]. Positively charged AMPs can selectively bind to negatively charged bacterial surface, cause the formation of pores and rupture of the bacterial membrane, the leakage of bacterial intracellular material and the collapse of the bacterial body, etc., inhibiting the growth of bacteria through the interruption of the integrity of the cell membrane [31,32,33,34]. As shown in time–killing assay (Figure 2), Q4-15a-1 exhibited bacteriostatic nature under low concentrations (0.5× and 1× MIC), while displaying dose-dependent and rapid bactericidal effect in higher concentrations (2× and 4× MIC). According to the results of Figure 3; Figure 4, Q4-15a-1 can interact with MDR ETEC to cause damage to the cell membrane and enter the cell in a very short time. We therefore assumed that the bactericidal properties of Q4-15a-1 may be attributed to the rapid destruction of the bacterial cell membrane of MDR ETEC.

Biofilm is a microbial community that adheres to the surface of various substances and encapsulates themselves in an autologous extracellular matrix. It is a critical issue to manage biofilms in clinical treatment [35]. The matrix of the biofilm plays an important role in the mechanism of resistance to antibiotics, which constitutes a barrier to delay or prevent the interaction of antibiotics with microbial cells, depending on the extracellular polymeric substances and the charge of antimicrobial agents. The latter can be isolated or repelled, thereby reducing its effect on biofilms [36,37], so it usually requires a four-times higher dosage to inhibit biofilms than that of planktonic bacteria [38]. In addition, the usual method applied for evaluating antibiofilm activity of drugs is to use crystal-violet to stain biofilms. However, crystal-violet staining can only stain bacterial cells and extracellular matrix, such as polysaccharides and proteins, so it cannot distinguish between living and dead cells [39]. In this study, we used MBEC method instead, by measuring the number of viable bacteria cells remaining in the biofilm after drug treatment with a plate count method can obtained data more closely related to the actual status of biofilms. According to research by Feng et al. [40], the use of 32 and 64 μg/mL of AMP LL37 has an inhibitory effect on two *Acinetobacter baumannii* biofilms. In this study, when MIC (4 μg/mL) level of AMP Q4-15a-1 was applied to treat ETEC biofilm for 24 h, the remaining viable biofilm bacteria was around 1.32%. Only four-times MIC (16 μg/mL) of Q4-15a-1 can remove 99.9% of ETEC biofilm, indicating that Q4-15a-1 has good anti-biofilm potential.

The colonization of ETEC in the porcine small intestine is primarily mediated by fimbria which confer the ability to attach to receptors on the enterocytes [25]. The ETEC causing neonatal colibacillosis mostly carry the fimbria F4 (k88), F5 (k99), F6 (987P), or F41, while that of post-weaning diarrhea carry F4 (k88) and F18 [41]. In this study, we intended to establish an infection model using two porcine small intestinal epithelial cell lines (IPEC-1 and IPEC-J2). The MDR ETEC strain used in this study has been determined to be F18-ciliated. According to a previous report indicating that IPEC-1 is more susceptible to F18 (K88) ciliated ETEC infection, and is less likely to adhere to IPEC-J2 cell lines [25,26], IPEC-1 cell line was selected for the following adherence assay instead of IPEC-J2. According to the results shown in Figure 7, when Q4-15a-1 and ETEC were added to IPEC-1 cells at different concentrations and co-cultured for 3 h, the number of ETEC adhered to IPEC-1 cells was significantly reduced in a dose-dependent manner. When ETEC were co-cultured with Q4-15a-1 of more than 32 μg/mL, the adherence of ETEC to IPEC-1 cells was completely inhibited. It is reasoned that the decreased adherence rate may be related to the good antibacterial effect of Q4-15a-1. A previous study reported that the peptide histatin-5 isolated from saliva reduces the number of ETEC adhesion to human colorectal cancer cell Caco-2 by blocking the special CFA/I cilia on ETEC [42]. It remains unclear whether AMP Q4-15a-1 may also block the attachment of ETEC to IPEC-1 cells by other strategies.

In summary, the present work was to explore the antibacterial and anti-biofilm activities of our self-designed novel AMPs against MDR strains of ETEC using MIC, MBC, and MBEC assays. Furthermore, we also established an MDR ETEC infection model with intestinal porcine epithelial cell-1 (IPEC-1). Our findings revealed that AMP Q4-15a-1 showed minimal cytotoxicity in the IPEC-1 cell line, while it exerted strong antimicrobial activity on MDR ETEC and significantly reduced its adhesion to IPEC-1 cells. To the best of our knowledge, this is the first report describing the antimicrobial efficacy of AMPs against MDR ETEC. Findings provided in this study could help us to understand the possible reaction mechanism of our novel AMPs and may facilitate the development of promising antibacterial candidate for treatment of weaned piglets infected by MDR ETEC.

## 4. Materials and Methods

### 4.1. Bacterial Strains and Culture Conditions

The MDR ETEC strain used in this study was a clinical isolate kindly provided by our co-author, Dr. Hui-Wen Chang from Department of Veterinary Medicine and Graduate Institute of Molecular and Comparative Pathobiology, School of Veterinary Medicine, National Taiwan University. It was confirmed to be resistant to several conventional antibiotics, including penicillins (ampicillin, augmentin, penicillin, and piperacillin), aminoglycosides (gentamicin, kanamycin, streptomycin, and tobramycin), β-lactam/β-lactamase inhibitor combinations (amoxicillin and clavulanate), cephalosporins (cefadroxil, cefixime, ceftazidime, cefuroxime, cephalothin, and cephazolin), fluoroquinolones (ciprofloxacin, enrofloxacin, flumequine, and ofloxacin), phenicols (chloramphenicol), quinolones (nalidixic acid and norfloxacin), tetracyclines (tetracycline, minocycline, and doxycycline), while moderately susceptible to nitrofurantoin and polymyxin B. Bacteria were cultured freshly for every experiment by cultivation from frozen stock at 37 °C for 12–14 h in Trypton Soy Broth (TSB, Oxoid, Basingstoke, UK). Glycerol stocks (20%, *v*/*v*) were maintained at −86 °C for long-term storage.

### 4.2. AMPs and Their Antibacterial Activity

AMPs used in the current study (summarized in Table 1) were originated from the most potent one, GW-Q4, selected from those designed as described in our previous work [12], with further truncation and modifications. AMPs were synthesized by Kelowna International Scientific Inc. Taiwan and purified to 95% by HPLC. Mass spectrometric analysis was also performed to confirm the molecular mass of all peptides (data not shown). The antimicrobial activities of AMPs were confirmed by a minimal inhibitory concentration (MIC) susceptibility test according to Chou et al. [12]. Briefly, MIC was determined by incubating in 135 µL of a final inoculum of 10^4^ CFU/mL bacterial suspension with various concentrations of 15 µL AMP solution (0–64 µg/mL) tested in the 96-well microtiter plates. Cultures were examined for growth after 48 h incubation at 37 °C, and the absorbance at 600 nm was measured. The MIC value was defined as the lowest concentration of AMP that completely inhibits visible bacterial growth after 48 h incubation.

### 4.3. In Vitro Time–Kill Curve Assay

The time–kill curve assay was also used to evaluate the antibacterial activities of AMPs against MDR ETEC. Overnight bacterial culture was harvested at 3 × 10^8^ CFU/mL into 30 mL of TSB and grown to OD_600nm_ = 0.2. The initial inoculum was treated with AMP Q4-15a-1 at concentrations of 0.5–4× MIC during 0–210 min. Samples collected every 30 min were diluted, plated on TSA agar plates, incubated for 12–14 h at 37 °C and counted. The CFU was determined and the percent killing of AMP Q4-15a-1 was calculated. Experiments were measured in triplicate and the results were exhibited as mean log (CFU/mL) ± SD.

### 4.4. Antibiofilm Assay

Antibiofilm assay was performed according to previous study [24] with slight modifications based on a published protocol using the Calgary biofilm device (Innovotech, Edmonton, AB, Canada). Briefly, MDR ETEC strain was incubated in TSB and cultures were diluted in the same medium to achieve a concentration of 10^5^ CFU/mL. Next, diluted bacterial suspension (150 µL) was added to the sterile 96 peg-lids on which biofilm cells can build up. Negative control lanes were prepared by adding 150 µL TSB to wells, and then the pegs were incubated for 24 h under rotation of 110 rpm at 37 °C to allow biofilm formation on the pegs. The pegs were rinsed twice with phosphate-buffered saline (PBS) to remove planktonic cells as a washing step. Each peg-lid was then transferred into a ‘challenge 96-well microtiter plate’ containing 200 µL of different AMP Q4-15a-1 concentrations and the peg-lids containing the biofilms were incubated for 24 h at 37 °C under rotation at 110 rpm. After biofilm treatment with the challenge plate, the biofilms were re-washed twice with PBS then transferred into the recovery plate for 12 h, then monitored the absorbance at 600 nm using a multimode microplate reader. The minimum biofilm eradication concentration (MBEC) value is defined as the minimum concentration needed to inhibit the re-growth of biofilms after 24 h of AMP treatment. Additionally, the biofilms were also assessed for their minimum bactericidal concentration (MBECb), which is defined as the lowest concentration able to eradicate 3log^10^ of the viable microorganisms in a biofilm (99.9% killing) after 12 h of incubation in recovery plates using the colony count method.

### 4.5. Fluorescent Staining and Confocal Microscopy

To monitor the cellular distribution of Q4-15a-1 in MDR ETEC, bacterial culture in the absence or presence of FITC-labeled Q4-15a-1 of 16 μg/mL were incubated at 37 °C for 10, 20, 30, and 60 min. We transferred 10 µl of bacterial solution to a new microcentrifuge tube with 100 µL of FM 4–64 (Invitrogen, Thermo Fisher Scientific, Waltham, MA, USA, 100 μg/mL, a red fluorescent capable of staining bacterial cell membrane) and incubated on ice for 1 min, then centrifuged at 10,000× *g* for 1 min to remove the staining solution. 50 µL of DPBS was added to resuspend the bacterial cells and 3.5 µL of bacterial solution and 5 µL of mounting gel were mixed on the slide glass and cover with a coverslip, then subjected to confocal microscopic observation on Olympus IX-81 optical microscope, (Olympus, Tokyo, Japan).

To further verify whether Q4-15a-1 can cause bacterial cell membrane damage of MDR ETEC, we utilized two fluorescent DNA intercalators, Hoechst 33,342 (Pierce Biotech, Thermo Fisher Scientific, Rockford, IL, USA, blue fluorescent) and Propidium iodide (PI, MilliporeSigma, Munich, Germany, red fluorescent). Bacterial culture in the absence or presence of Q4-15a-1 of 16 μg/mL were incubated at 37 °C for 10, 20, 30, and 60 min. We transferred 10 µL of bacterial solution to a new microcentrifuge tube with 100 µl of Hoechst 33,342 (20 μg/mL) and PI (20 μg/mL) and staining for 30 min, then centrifuged at 10,000× *g* for 1 min to remove the staining solution. 50 µL of DPBS was added to resuspend the bacterial cells and 3.5 µL of bacterial solution and 5 µL of mounting gel were mixed on the slide glass and cover with a coverslip, then subjected to confocal microscopic observation on Olympus IX-81 optical microscope (Olympus, Tokyo, Japan).

### 4.6. Transmission Electron Microscopy (TEM)

The TEM procedures followed the protocols of a previous publication [43]. Briefly, ETEC were concentrated by centrifugation (Beckman Coulter, Avanti J-20XP, JA-25.50, Palo Alto, CA, USA) at 1700× *g* for 10 min at 4 °C. The pellet was resuspended in PBS to a final concentration of 2 × 10^9^ CFU/mL. The bacterial suspension (5 mL) was mixed with a freshly prepared AMP Q4-15a-1 solution in PBS to a final concentration of 16 or 32 μg/mL (4× or 8× MIC). The samples were kept overnight at 4 °C in fixative (2.5% glutaraldehyde in 1×PBS; pH: 7.4) and there after postfixed in 1% OsO4 in the same buffer. After dehydration, in graded ethanol, the samples were finally embedded in Spurr resin (Spurr Low Viscosity Embedding Kit; EMS ^®^). Ultrathin sections were cut on a Leica^®^ Ultracut UC7 Ultramicrotome (Leica Microsystems, Vienna, Austria) equipped with a diamond knife, stained with uranyl acetate and lead citrate then examined in a FEI Tecnai G2 F20 S-TWIN TEM (FEI, Hillsboro, OR, USA) at 120 kV in the Institute of Cellular and Organismic Biology, Academia Sinica.

### 4.7. Cell Lines and Culture Conditions

The IPEC-1 and IPEC-J2 cell lines were kindly provided by Dr. Je-Ruei Liu from Institute of Biotechnology and Department of Animal Science and Technology, National Taiwan University. The culture conditions and protocols were according to Koh et al. [25] with slight modifications. Both IPEC-1 and IPEC-J2 cells were seeded on 9-cm cell culture dishes and were cultured and maintained in Dulbecco’s modified Eagle medium (DMEM)/nutrient mixture F-12 (GeneDireX, Taipei, Taiwan) supplemented with 10% fetal bovine serum (FBS; Hyclone, Cytiva, Washington DC, USA). The cultures were maintained in a humidified incubator in an atmosphere of 5% CO_2_ at 37 °C. After 4 to 5 days of culturing, both cell lines became confluent. Cell monolayers were washed with 3–5 mL Dulbecco’s phosphate buffered saline (DPBS, Amresco, Solon, OH, USA) and trypsinized with 1× trypsin-EDTA. The detached cells were pelleted at 200× *g* for 5 min, re-suspended in antibiotic-free medium, and used for subsequent adherence assays. The continuous cultures of both cell lines were maintained by seeding 9-cm culture dishes at 1:3 ratios at each passage.

### 4.8. Cell Viability Assay

The viability of both IPEC-1 and IPEC-J2 cells after treated with AMP Q4-15a-1 was evaluated using 3-(4,5-dimethylthiazol-2-yl)-2,5-diphenyltetrazolium bromide (MTT, Amresco, Solon, OH, USA) assays performed in triplicate in three independent experiments. Briefly, cells were plated at a density of 10^4^ cells/well in 96-well plates, and were permitted to adhere for 24 h then washed with phosphate buffered saline (PBS, Amresco, Solon, OH, USA). Solutions were always prepared freshly by dissolving 1× PBS or AMP Q4-15a-1 in culture medium and added to both cell lines. After 24 h of exposure, the peptide containing medium was removed, washed with PBS and replaced by fresh medium. The cells in each well were then incubated in culture medium with 0.5 µg/mL MTT for 2 h. After the media were removed, 200 µL of DMSO were added to each well. Absorbance at 570 nm of the maximum was detected by a multimode microplate reader SpectraMax M2 (Molecular Devices, San Jose, CA, USA). The viability of DMSO-treated cells was considered as 100%.

### 4.9. Bacterial Adherence Assays

The adherence assay procedure was as described in a previous study [44]. Briefly, IPEC-1 cells were adjusted to 2 × 10^5^ cells/mL and 1 mL of cell culture fluid was added to 24-well plate, cultured in a 37 °C incubator with 5% CO_2_ for 24 h until the cells were attached. After removing the supernatant waste liquid, cells were washed with warm DPBS, added 1 mL of cell culture medium, followed by the addition of MDR ETEC of MOI 5 (10^6^ CFU). After infection, we carefully washed the cells twice with warm DPBS to remove unattached ETEC suspension, added 100 µL of 0.1% triton X -100 (J.T.Baker, Thermo Fisher Scientific, Rockford, IL, USA) to the cells for 10 min, then added 900 µL of TSB to dilute the bacterial culture. Diluted bacterial cultures were then subjected to plate count on TSA for 12 to 14 h, and extent of bacterial binding to IPEC-1 cells was determined. In experiments monitoring the effect of AMP on the bacterial adherence to IPEC-1 cells, different concentrations of Q4-15a-1 (4–128 μg/mL) were added to the MDR ETEC culture before infection. Each adherence assay was repeated three times or more to confirm the consistency of results.

### 4.10. Statistical Analysis

Data were expressed as mean values plus standard deviations. Differences in mean Log (CFU/mL) of surviving colonies between untreated and each peptide group (at concentrations of 1×, 4×, and 8× MIC) and cell viability and adherence rate in the study of inhibitory effect of AMP against the adherence of MDR ETEC to IPEC-1 cells were performed with Prism 6 statistical analysis software (Graph-Pad, San Diego, CA, USA). One-way ANOVA combined Bonferroni’s multiple comparison test was used to specify the differences between groups. A *p*-value of < 0.05 was considered statistically significant. *p*-value: *, ** < 0.05; *** < 0.0001.

## 5. Conclusions

The present study was to explore the antibacterial and anti-biofilm activities of our self-designed novel AMPs against MDR strains of ETEC using MIC, MBC, and MBEC assays. Furthermore, we also established an MDR ETEC infection model with intestinal porcine epithelial cell-1 (IPEC-1). Our findings in this study revealed that AMP Q4-15a-1 showed minimal cytotoxicity in the IPEC-1 cell line, while it exerted strong antimicrobial activity on MDR ETEC and significantly reduced its adhesion to IPEC-1 cells. Multiple strategies are now under investigation to further improve the antimicrobial efficacy and stability of AMPs against MDR pathogens. Findings provided in this study could help us to understand the possible reaction mechanism of our novel AMPs and may facilitate the development of promising antibacterial candidate for treatment of weaned piglets infected by MDR ETEC.

## Figures and Tables

**Table 1:** Structural parameters and molecular weight of antimicrobial peptides (AMPs) used in the current study.

Peptide	Amino Acid Sequence ^a^	Molecular Weight	Net Charge	Hydrophobicity ^b^
GW-Q4	**GANAA**KKFATIAKKFINYLW	2255.69	+4	−0.85
GW-Q4a	**GANAA**KKFATIAKKFINYLW-NH_2_	2254.73	+5	−0.85
Q4-15	KKFATIAKKFIN**Y**LW	1871.30	+4	−1.12
Q4-15a	KKFATIAKKFIN**Y**LW-NH_2_	1870.33	+5	−1.12
Q4-15-1	KKFATIAKKFIN**K**LW	1836.31	+5	−2.24
Q4-15a-1	KKFATIAKKFIN**K**LW-NH_2_	1835.33	+6	−2.24
Q4-15-2	KKFATIAKKFIN**W**LW	1894.35	+4	−0.77
Q4-15a-2	KKFATIAKKFIN**W**LW-NH_2_	1893.37	+5	−0.77

^a^ Boldface type indicates amino acid residues truncated or substituted in the Q4 derivatives. ^b^ Hydrophobicity was calculated using the consensus value for each amino acid residue as described [23].

**Table 2:** Minimal inhibition concentration (MIC), minimal hemolysis concentration (MHC) and therapeutic index (TI) values of AMPs against multidrug-resistant *Escherichia coli* (MDR ETEC).

Peptide	Amino Acid Sequence ^a^	MIC (μg/mL)	MHC (μg/mL)	TI (MHC/MIC)
GW-Q4	**GANAA**KKFATIAKKFINYLW	32	128	4
GW-Q4a	**GANAA**KKFATIAKKFINYLW-NH_2_	8	32	4
Q4-15	KKFATIAKKFINYLW	8	128	16
Q4-15a	KKFATIAKKFINYLW-NH_2_	4	16	4
Q4-15-1	KKFATIAKKFIN**K**LW	8	256	32
Q4-15a-1	KKFATIAKKFIN**K**LW-NH_2_	4	256	64
Q4-15-2	KKFATIAKKFIN**W**LW	4	32	8
Q4-15a-2	KKFATIAKKFIN**W**LW-NH_2_	2	16	8

^a^ Boldface type indicates amino acid residues truncated or substituted in the Q4 derivatives.

**Table 3:** Viable biofilm cells of multidrug-resistant enterotoxigenic *Escherichia coli* (MDR ETEC) after treated with Q4-15a-1 for 24 h.

Peptide Concentration (μg/mL)	Viable Biofilm Cells (%)	± SD
128	0	0
64	0	0
32	0	0
16	0.003	0.001
8	0.162	0.021
4	1.323	0.107

**Table 4:** Antibiofilm activity of Q4-15a-1 against MDR ETEC.

	Peptide Concentration (μg/mL)
MBEC ^a^	15.93 ± 4.07
MBECb ^b^	16

^a^: MBEC is defined as the minimum concentration needed to inhibit the re-growth of biofilms after 24 h of peptide treatment [24]. ^b^: MBECb is defined as the lowest concentration able to eradicate 3log_10_ of the viable microorganisms in a biofilm (99.9% killing).
